# Alterations in Circulating miRNA Levels following Early-Stage Estrogen Receptor-Positive Breast Cancer Resection in Post-Menopausal Women

**DOI:** 10.1371/journal.pone.0101950

**Published:** 2014-07-08

**Authors:** Annette R. Kodahl, Pernille Zeuthen, Harald Binder, Ann S. Knoop, Henrik J. Ditzel

**Affiliations:** 1 Department of Oncology, Odense University Hospital and University of Southern Denmark, Odense, Denmark; 2 Department of Surgery Z, Odense University Hospital, Odense, Denmark; 3 Institute of Medical Biostatistics, Epidemiology and Informatics (IMBEI), Johannes Gutenberg University, Mainz, Germany; 4 Department of Oncology, Rigshospitalet, Copenhagen University Hospital, Copenhagen, Denmark; 5 Department of Cancer and Inflammation, Institute of Molecular Medicine, University of Southern Denmark, Odense, Denmark; SAINT LOUIS UNIVERSITY, United States of America

## Abstract

**Introduction:**

Circulating microRNAs (miRNAs) exhibit remarkable stability and may serve as biomarkers in several clinical cancer settings. The aim of this study was to investigate changes in the levels of specific circulating miRNA following breast cancer surgery and evaluate whether these alterations were also observed in an independent data set.

**Methods:**

Global miRNA analysis was performed on prospectively collected serum samples from 24 post-menopausal women with estrogen receptor-positive early-stage breast cancer before surgery and 3 weeks after tumor resection using global LNA-based quantitative real-time PCR (qPCR).

**Results:**

Numbers of specific miRNAs detected in the samples ranged from 142 to 161, with 107 miRNAs detectable in all samples. After correction for multiple comparisons, 3 circulating miRNAs (miR-338-3p, miR-223 and miR-148a) exhibited significantly lower, and 1 miRNA (miR-107) higher levels in post-operative vs. pre-operative samples (p<0.05). No miRNAs were consistently undetectable in the post-operative samples compared to the pre-operative samples. Subsequently, our findings were compared to a dataset from a comparable patient population analyzed using similar study design and the same qPCR profiling platform, resulting in limited agreement.

**Conclusions:**

A panel of 4 circulating miRNAs exhibited significantly altered levels following radical resection of primary ER+ breast cancers in post-menopausal women. These specific miRNAs may be involved in tumorigenesis and could potentially be used to monitor whether all cancer cells have been removed at surgery and/or, subsequently, whether the patients develop recurrence.

## Introduction

Breast cancer is the second leading cause of cancer death among women in Europe and North America. Although five-year survival rates for localized breast cancer is over 95%, it is only 27% with distant metastasis [1]. After completion of adjuvant therapy, follow-up care focuses on detecting recurrent disease in the hope of improving long-term survival. However, no ideal biomarkers have yet been found for this purpose. Breast cancer staging is based on tumor size, lymph node status, and the presence or absence of metastatic disease. Currently, axillary lymph node status in stage I and II is the strongest prognostic factor, but 25% of patients without axillary lymph-node metastasis will have a systemic relapse. [2]. This can be explained by the presence of systemic micrometastatic disease. Breast cancer follow-up for the purpose of early detection of local or metastatic recurrence is predominantly based on clinical examination and mammography, and previously suggested tumor markers, such as CA 15.3, CA 27.29 and carcinoembryonic antigen (CEA), are not recommended in the routine follow-up after initial treatment [3].

MicroRNAs (miRNAs) are small, 18–25 nucleotide long, single-stranded, non-coding RNA molecules that down-regulate the translation of messenger RNA (mRNA) in both animal and plant cells [4]. Their biogenesis begins in the nucleus, where precursor miRNAs are synthesized from genomic DNA using RNA polymerase II. The precursor miRNA is then cleaved by RNAse III endonuclease to precursor miRNA with a characteristic stem-loop structure. The precursor miRNA leaves the nucleus by the Exportin-5 receptor that mediates the transport of proteins from the nucleus to the cytoplasm [5]. In the cytoplasm, the loop region is cut off and a miRNA duplex remains. One strand of the duplex is the mature miRNA, which becomes part of a protein complex (RNA-induced silencing complex, RISC), while the second string is often degraded. RISC blocks the translation of mRNA by imperfect match to the target mRNA (often seen in animal cells) or leads to degradation of mRNAs by perfect match (typically seen in plants) [6]. It has been suggested that at least 30–60% of human genes are directly targeted by miRNAs [7], and thus miRNAs can control a wide array of physiological and pathological processes, including development, differentiation, cellular proliferation, programmed cell death, oncogenesis and metastasis [6], [8]–[10]. Circulating miRNAs have been proposed as promising novel biomarkers for cancer and several other diseases [11], which can be measured without invasive biopsies. MiRNAs are easily measured in blood samples and show remarkable stability in plasma and serum even after extended storage at room temperature or multiple freeze/thaw cycles [12]. In the circulation, they are most likely protected from RNAse degradation by their binding to protein-complexes [13]. MicroRNAs might enter the circulation either by passive leakage from apoptotic or necrotic cells or by active secretion of microvesicle-free miRNA or miRNA-containing microvesicles. While the half-life of tumour-associated miRNAs in the blood is undefined, it has previously been suggested to be less than 14 days [14].

Since late 2009, several groups reported on circulating miRNAs as markers for breast cancer detection [14]–[24]. Most of these studies focused on comparing circulating levels of miRNAs in cancer patients to that of healthy controls, while only few studies investigated alterations in miRNA levels after surgery of early-stage breast cancer [14], [22], [23], [25]. Two of these studies investigated the altered levels of a few pre-selected miRNAs [14], [23]. Leidner et al. performed global miRNA expression analysis on plasma samples from 20 breast cancer patients, 20 healthy controls and 20 breast cancer post-resection cases. However, because the pre-and post-resection samples were not from the same patients, drawing clear conclusions from this study is difficult [22]. Only one previous study performed global miRNA analysis on paired pre- and post-surgery plasma samples from patients with early-stage breast cancer, and employed the same experimental platform as used in our study [25].

Here, we report our study aimed at investigating alterations in the level of circulating miRNAs following surgery of primary breast cancers using prospectively collected serum samples from 24 post-menopausal women with estrogen receptor (ER)-positive early-stage breast cancer before surgery and 3 weeks after tumor resection. A panel of 4 circulating miRNAs was found to exhibit significantly altered levels using global LNA-based quantitative real-time PCR (qPCR).

## Materials and Methods

### Ethics Statement

The study was performed in accordance with the Declaration of Helsinki and approved by the regional ethics committee (Project-ID: S-20100132) and the Danish Data Protection Agency (ID: 2008-58-0035). Written informed consent was obtained from all participants in this study.

### Sample and data collection

The study population was obtained from a prospective study performed at a single center cancer hospital. A total of 137 patients with primary operable breast cancer were recruited from 5/2011–2/2012. All participants were asked to donate a blood sample prior to surgery. No patients received any treatment, such as neo-adjuvant chemotherapy or radiation therapy, prior to surgery. Patients eligible for adjuvant treatment were further asked to donate a blood sample at the time of the initial visit at the Department of Oncology at Odense University Hospital (∼3 weeks post-surgery) prior to any medical or radiation treatment. A total of 95 patients had paired blood samples before and after surgery, and 24 patients from this cohort were included in the study based on the following criteria: Post-menopausal patients with an ER-positive and HER2-negative primary operable ductal carcinoma, 2 or less axillary lymph node metastasis and a tumor diameter of 20 mm or less (Table 1). Histopathological diagnosis was confirmed after surgical resection of the tumors. Serum was prepared within one hour of sample collection after centrifugation (2000×g; 10 min at 20 °C) and immediately stored at −80 °C.

**Table 1:** Characteristics of the study participants.

Parameters	Patients (%)
Total	24
Median age, yr	66
Range	54–81
Post-menopausal	24 (100)
ER+	24 (100)
Median tumor size, mm	12
Range	5–16
HER2+	0
Nodal status	
0	17 (75)
1–2	7 (25)
Ductal carcinoma	24 (100)
Treatment	
Lumpectomy	20 (83)
Mastectomy	4 (17)

The data discussed in this publication have been deposited in NCBI's Gene Expression Omnibus [26] and are accessible through GEO Series accession number GSE57661 (http://www.ncbi.nlm.nih.gov/geo/query/acc.cgi?acc=GSE57661).

### RNA extraction and miRNA real-time quantitative PCR

Total RNA was extracted from 250 µl of serum using the miRNeasy Mini Kit (Qiagen, Hilden, Germany) according to manufacturer's instructions. The standard protocol was modified according to Exiqońs application note “RNA Purification from Blood Plasma & Serum” (http://www.exiqon.com/ls/Documents/Scientific/serum-plasma-RNA-isolation.pdf) by adding 1.25 µl MS2 carrier RNA (Roche) to the QIAzol Reagent prior to RNA purification to maximize the yield and minimize purification efficiency variation [27]. Total RNA was eluted by adding 50 µL of RNase-free water to the membrane of the spin column and incubating for 1 min before centrifugation at 15,000×g for 1 min at room temperature. The RNA was immediately stored at −80 °C. RNA (4 µl) was reverse-transcribed in 20 µl reactions using the miRCURY LNA Universal RT microRNA PCR, polyadenylation and cDNA synthesis kit (Exiqon). cDNA was diluted 50× and assayed in 10 µl PCR reactions according to the manufacture's protocol for miRCURY LNA Universal RT microRNA PCR; each microRNA was assayed once on microRNA Ready-to-Use PCR Serum/Plasma Panel (Exiqon, Vedbaek, Denmark). Negative controls, excluding the template from the reverse transcription reaction, were profiled similarly to the test samples. As technical controls, an RNA spike-in (Sp6) was added in the reverse transcription reaction to evaluate the RT reaction. In addition, a DNA spike-in (Sp3) was included in triplicates on all panels as an indicator of possible inhibitions at the qPCR level. The amplification was performed by a LightCycler 480 Real-Time PCR System (Roche, Basel, Switzerland) in 384-wells. The amplification curves were analyzed using the Roche LC software, both for determination of Cp (by the 2nd derivative method) and for melting curve analysis. All assays were inspected for distinct melting curves and the Tm was confirmed to be within known assay specifications. Any data points that showed multiple peaks were excluded from the data set. Assay efficiencies were determined by analysis of the amplification curves using algorithms similar to the LinReg software package. The efficiencies ranged between 1.8 and 2.1. Individual reactions with efficiencies <1.6 were excluded from the dataset.

### Sample quality

To obtain an estimate of the data quality for each sample, we compared the level of microRNAs (number of microRNAs detected as well as the average Cp for each sample) in all samples. Very similar data was obtained, suggesting that the samples were of similar quality and processed reproducibly.

### Normalization and data analysis

Data points with cycle thresholds greater than 37 or less than 5 cycles lower than the cycle threshold for the negative control were excluded from the data analysis. Two normalization approaches were used. Initially, normalization was performed based on the average of the assays detected in all samples, as this has shown to be the best normalization for qPCR studies involving numerous assays [28]. For the present study, this included 107 assays.

The formula used to calculate the normalized Cp values was: Normalized Cp  =  average Cp (n = 107) – assay Cp (sample). However, for experiments involving just a few assays, endogenous controls must be selected, and for this purpose we used the NormFinder software to select possible normalization candidates for a potential validation study [29]. Two miRNAs (miR-148b and miR-30 d) were selected. This method of normalization was also used to test for reproducibility in the Cookson data set [25].

### Evaluation for potential hemolysis of the blood samples

To determine whether hemolysis of the blood samples had occurred during sample preparation and thus released excess blood component-related miRNAs, the ratio between the expression levels of two miRNAs was determined; miR-451, expressed in red blood cells and miR-23a, relatively stable in serum and plasma and not affected by hemolysis. It has been suggested that a delta Cp (miR-23a – miR-451) of more than five is an indicator of possible erythrocyte miRNA contamination, whereas a delta Cp of 7–8 or more indicates high risk of hemolysis [30]. We re-ran the analysis excluding 5 sample pairs in which the post-operative sample had a delta Cp larger than 8. Overall, the analysis did not change much and we are therefore confident that the differences between pre- and post-operative samples were not affected by hemolysis in the sample set.

### Statistical analysis

Paired, two-tailed, t-tests were used to identify miRNAs significantly changed between pre- and post-operative samples. Significant values were corrected for multiple testing using the Bonferroni method. The raw data set from a similar study using the same qPCR platform [25] were kindly provided by the corresponding author and after using the two different normalization approaches, we compared the miRNAs that were found to be significantly altered in the post-resection samples of our study to the that of Cookson et al. [25]. The proposed biomarkers of Cookson et al. (let-7b, let-7g and miR-18a) were also evaluated in our data set. All p-values were two-tailed, and p-value ≤0.05 was considered statistically significant. All statistical calculations were performed using the statistical environments R 3.0.2 and STATA 13.1.

## Results

### Alterations in levels of circulating miRNAs following surgery

Global miRNA analysis was performed on serum from 24 postmenopausal patients with ER-positive and HER2-negative early-stage breast cancer prior to tumor resection as well as 3 weeks after surgery using LNA-based qRT-PCR (Table 1). Numbers of miRNAs detected in the samples ranged from 142 to 161, with 107 miRNAs detectable in all samples. The mean number of detectable miRNAs in both sample groups was 153, ranging from 143 to 161 in the pre-operative and 142 to 160 in the post-operative samples. There was no difference in mean miRNA levels between samples taken before and after surgery (p = 0.51). No miRNAs were consistently undetectable in the post-operative compared to pre-operative samples.

**Figure 1:**
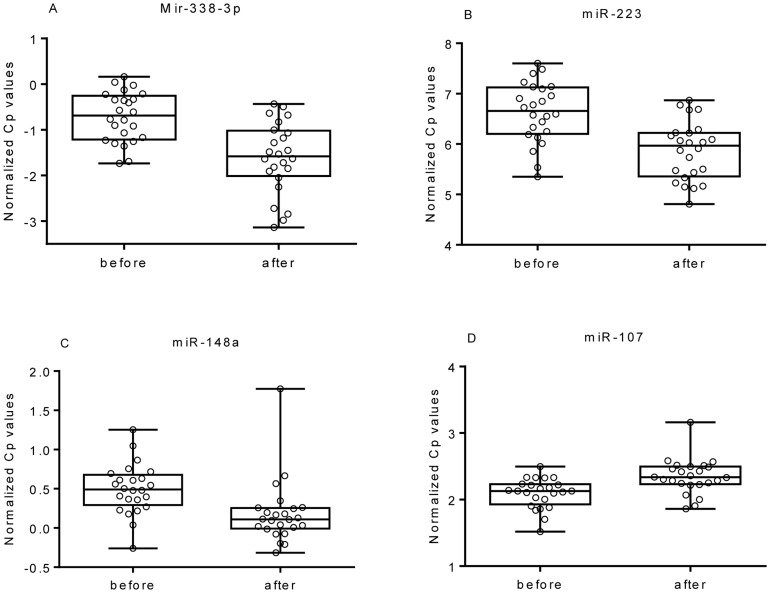
Box plots. Box plots showing the expression levels of (A) miR-338-3p, (B) miR-223, (C) miR-148a and (D) miR-107 before (n = 24) and after (n = 24) resection of early-stage breast cancer. Each circle represents a sample value.

**Figure 2:**
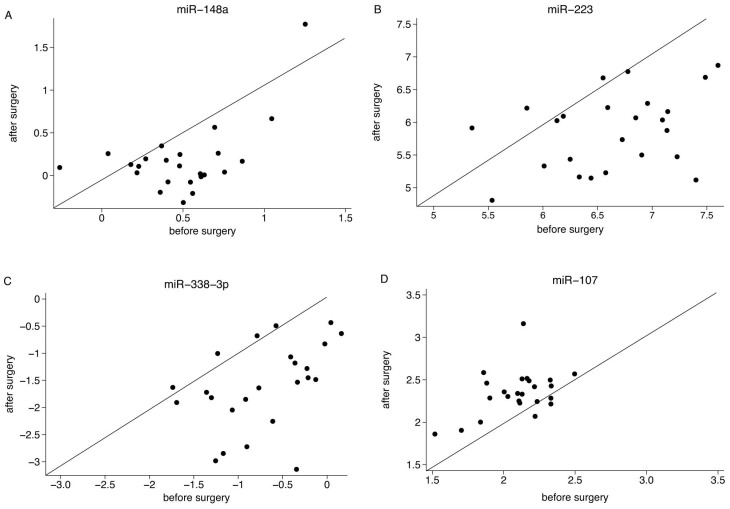
Scatter plots. Scatter plots showing the corresponding expression levels of (A) miR-148a, (B) miR-223, (C) miR-338-3p and (D) miR-107 for the paired pre- and post resection samples for each early-stage breast cancer patient (n = 24).

To evaluate the alteration in levels of each miRNA in the post-operative vs. pre-operative samples, all sample Cp-values were normalized to the average Cp-value detected in all samples (n = 107). Among the 174 miRNAs analyzed, 28 had an uncorrected p-value ≤0.05 when testing for differential expression in serum before vs. after surgery (Table 2). After correction for multiple comparisons, 3 circulating miRNAs (miR-338-3p, miR-223 and miR-148a) exhibited significantly lower, and 1 miRNA (miR-107) exhibited higher, levels in the post- vs. pre-operative samples (Figure 1). The most consistently down-regulated miRNAs (miR-223 and miR-148a) were down-regulated in 21 of the 24 post-operative samples (87%), whereas miR-338-3p was down-regulated in 20 of the 24 post-operative samples (83%). The corresponding values for the paired pre- and post resection samples for each patient are illustrated in Figure 2. MicroRNA-107 was up-regulated in 21 of the 24 patients (87%) following surgery. In addition to normalization to the mean Cp of all samples, we also analyzed the miRNA data using normalization to the two miRNAs identified using NormFinder (miR-148b and miR-30d). The results based on the two different normalization methods are provided in Table 3.

**Table 2:** Circulating microRNAs differentially expressed in the serum of patients with early-stage breast cancer before tumor resection compared to ∼3 weeks post-surgery.

miRNA	Mean ΔCp before surgery (N = 24)	Mean ΔCp after surgery (N = 24)	p-value	Bonferroni adjusted p-value
**miR-338-3p**	−0.72	−1.61	3.9E-06	0.000678
**miR-223**	6.63	5.87	1.22E-05	0.0021
**miR-107**	2.09	2.35	8.8E-05	0.0153
**miR-148a**	0.50	0.18	0.00025	0.0435
miR-140-5p	−1.60	−2.10	0.0006	0.1118
miR-223*	−2.62	−3.56	0.0010	0.1697
miR-424	1.21	0.86	0.0015	0.2687
miR-103	2.80	3.01	0.0016	0.2799
miR-23a	2.99	2.64	0.0021	0.3673
miR-197	−0.95	−1.42	0.0035	0.6168
let-7g	1.27	1.51	0.0047	0.8102
let-7i	0.20	0.40	0.0053	0.9148
miR-18a	−1.69	−1.48	0.0066	>1
miR-144	3.59	4.34	0.0069	>1
miR-24	3.20	2.90	0.0111	>1
miR-27a	2.44	2.19	0.0120	>1
miR-15b	2.13	2.01	0.0120	>1
let-7d*	−0.41	−0.69	0.0141	>1
miR-532-5p	−2.94	−2.62	0.0191	>1
miR-361-3p	−3.09	−3.48	0.0228	>1
miR-584	−3.62	−4.00	0.0262	>1
miR-30e	−1.83	−2.10	0.0329	>1
miR-23b	0.69	0.44	0.0352	>1
miR-29a	0.63	0.48	0.0396	>1
miR-29b	−2.24	−1.98	0.0422	>1
miR-29b-2*	−5.58	−5.15	0.0453	>1
miR-106a	2.59	2.79	0.0484	>1
miR-142-5p	0.12	−0.09	0.0499	>1

MiRNAs in bold pass Bonferroni correction.

**Table 3:** Significantly deregulated miRNAs after surgery using two different normalization approaches.

	Mean normalized (n = 107)	Normalized using miR-148b and miR-30d
Significantly down-regulated miRNAs after surgery	**338-3p**, **223**, **148a**, **140-5p**, 223*, 424, **23a**, **197**, **24**, **27a**, **584**, **30e**, **23b**, 15b, let-7d*, 361-3p, 29a, 142-5p	**338-3p**, **223**, **148a**, **140-5p**, 223*, 424, **23a**, **197**, **24**, **27a**, **584**, **30e**, **23b,** 425*, 28-3p, 92b
Significantly up-regulated miRNAs after surgery	**107**, **103**, **let-7g**, **let-7i**, **18a**, **144**, **532-5p**, 29b, 29b-2*, 106a	**107**, **103**, **let-7g**, **let-7i**, **18a**, **144**, **532-5p**, 29b-2*

MiRNAs in bold are regulated in the same direction using both normalization methods. Paired student's t-test (p<0.05).

### Comparison of our results with that obtained in an independent study using similar study design and miRNA qPCR platform

To investigate whether the altered circulating miRNAs found in our study also exhibited altered levels in an independent study using similar study design and the same qPCR platform, we compared the results from our study to that of Cookson et al. [25]. Table 4 presents the profiling results of the miRNAs with (uncorrected) p-value ≤0.05 from our study in the Cookson data set using the two different normalization approaches. From these 28 miRNAs, 10 were also significantly altered in the Cookson data when normalizing to the mean expression, and 5 when normalizing to miR-148b and -30d. Only 4 of these were significantly altered 2 weeks after surgery in the Cookson dataset, and the rest only after 6 months. However, all except 2 miRNAs (miR-30e and miR-15b) were altered in the opposite direction and only when normalizing to the mean levels. We also compared the 3 biomarkers proposed by Cookson et al. (let-7b, let-7g and miR-18b) in our study. As shown in Table 4, let-7g was significantly altered in our study, but in the opposite direction, and let-7b and miR-18b expression was not significantly altered after surgery in our study (data not shown).

**Table 4:** Top 28 candidate miRNAs from our current study compared in Cookson et al. dataset using two alternative normalization methods.

	Normalized to mean				Normalized to miR-148b and miR-30d			
	Kodahl et al.		Cookson et al.		Kodahl et al.		Cookson et al.	
miRNA	p-value	Change	p-value†	Change	p-value	Change	p-value	change
miR-338-3p	3.9E-06‡	decreased	ns		5.19E-06	decreased	ns	
miR-223	1.22E-05‡	decreased	ns		4.58E-06	decreased	ns	
**miR-107**	8.8E-05‡	increased	0.02	decreased*	0.0012	increased	0.027	decreased
**miR-148a**	0.00025‡	decreased	0.03	increased*	0.0058	decreased	ns	
miR-140-5p	0.0006	decreased	ns		1.39E-05	decreased	ns	
miR-223*	0.001	decreased	ns		0.0004	decreased	ns	
miR-424	0.002	decreased	ns		0.054		ns	
**miR-103**	0.002	increased	0.03	decreased*	0.005	increased	0.024	decreased
miR-23a	0.002	decreased	ns		0.002	decreased	ns	
miR-197	0.004	decreased	ns		0.009	decreased	ns	
**let-7g**	0.005	increased	0.03	decreased	0.030	increased	ns	
**let-7i**	0.005	increased	0.04	decreased	0.031	increased	0.015	decreased
**miR-18a**	0.007	increased	0.04	decreased*	0.032	increased	ns	
miR-144	0.007	increased	ns		0.005	increased	ns	
miR-24	0.011	decreased	ns		0.006	decreased	ns	
miR-27a	0.012	decreased	ns		0.006	decreased	ns	
**miR-15b**	0.012	decreased	0.01	decreased*	0.099		ns	
let-7d*	0.014	decreased	ns		0.072		ns	
**miR-532-5p**	0.019	increased	0.03	decreased	0.047	increased	0.044	decreased
miR-361-3p	0.023	decreased	ns		0.054		ns	
miR-584	0.026	decreased	ns		0.031	decreased	ns	
**miR-30e**	0.033	decreased	0.02	decreased*	0.029	decreased	ns	
miR-23b	0.035	decreased	ns		0.015	decreased	ns	
miR-29a	0.040	decreased	ns		0.2		ns	
**miR-29b**	0.042	increased	0.03	decreased	0.1		0.018	decreased
miR-29b-2*	0.045	increased	ns		0.011	increased	ns	
miR-106a	0.048	increased	ns		0.1		ns	
miR-142-5p	0.0499	decreased	ns		0.1		ns	

MiRNAs in bold are changed after surgery in both studies using at least one normalization method.

† 1-sided as reported by Cookson et al.

*only 6 months after surgery.

‡ Pass Bonferroni correction. ns: not significant.

## Discussion

We investigated changes in the levels of specific circulating miRNAs following tumor resection in a selected cohort of post-menopausal women with ER-positive early-stage breast cancer. We found 28 miRNAs with significantly altered levels after surgical removal of the tumor, and 4 of these passed the Bonferroni correction for multiple comparisons (miR-338-3p, miR-223, miR-148a and miR-107).

A major concern in this field of research is the lack of reproducible results, probably due to differences in study populations, sample material and preparation, platforms, miRNA extraction procedures, normalization techniques and statistical approaches. Only one previous study investigated the changes in miRNA expression following early stage breast cancer surgery using the same qPCR platform as we, thus allowing comparison of the two sets of data [25]. Comparison of our data with those of Cookson et al. [25], who also studied changes in circulating miRNA following tumor resection in early stage breast cancer patients, showed little overlap. Although we used the same LNA-based qPCR platform, the two studies differed in several aspects. The study by Cookson contained a mixture of patients with ER-positive (60%) and ER-negative (40%) tumors, two quite different subtypes that likely have led to differences in miRNA levels, particularly in the pre-surgery blood samples. A strength of our study is the use of a highly homogeneous sample group, which adds to the statistical power as well as minimizes differences in miRNA expression due to disease sub-grouping. This, however, may also be a drawback, since the results of this study might only be applicable to this specific subgroup of patients. Another difference between the study by Cookson et al. and ours is that they used plasma samples, while we used serum. Measurements of miRNAs in serum and plasma have previously been found to be highly correlated [12], [30], but factors such as sample handling, preparation and the use of different collection tubes in serum vs. plasma could affect the miRNA profile. Both we and the Cookson's group used two different normalization approaches; 1) normalization based on the average of the assays detected in all samples, and 2) normalization using endogenous controls. Cookson et al. normalized to miR-16 due to a previous study indicating relatively invariant levels of this particular miRNA [14]. However, in the study by Cookson et al., miR-16 was found to be one of the least suitable individual miRNAs for normalization. Of the 4 altered miRNAs in our study, 2 miRNAs (miR-107 and miR-148a) were also significantly altered in the Cookson dataset, but in the opposite direction.

Differences in the time of collection of the postoperative samples may impact miRNA expression levels. The half-life of circulating miRNAs remain undetermined, but it has previously been suggested to be less than two weeks [31]. In the study by Cookson et al., postoperative samples were collected two weeks and also 6 months after surgery [25]. It is not clear whether the circulating miRNA levels changes between 2 and 3 weeks post-surgery, but it seems likely as miRNAs associated with subclinical inflammation due to the surgery, as well as miRNAs associated with the tumors would gradually decline. Evaluation of circulating miRNA levels several months after surgery is difficult, since most patients at this point have been exposed to different kinds of adjuvant treatment that could potentially affect miRNA levels.

A few other studies also investigated changes in circulating miRNA levels after breast cancer surgery [14], [22], [23]. Surprisingly, we were not able to support any of these previously-published associations between breast cancer and the expression of circulating miRNAs, including let-7a and miR-195 [14], miR-16, miR-21 and miR-451 [23] and miR-708*, miR-92b* and miR-568 [22].

Ng et al. investigated changes in circulating miRNA expression after tumor resection [23], but their investigation included only the expression levels of a few preselected miRNAs (miR-16, miR-21 and miR-451) in 15 pre- and post-resection samples. Likewise, Heneghan et al. investigated changes in expression of a few preselected miRNAs (miR-10b, miR-21, miR-145, miR-155, miR-195 and let-7a) following breast cancer surgery [14]. Thus, these studies might have missed other miRNAs with changed expression levels after surgery. Leidner et al. compared the global expression of circulating miRNAs in plasma samples from 20 pre-and 20 post-resection breast cancer cases [22], however, the pre- and post-resection samples were not from the same patient material, which means the observed difference in the expression levels of circulating miRNAs might be due to differences in patient material and not to tumor resection. Moreover, none of the above studies used the same qPCR platform as we, making direct comparisons of data sets problematic.

The role of miR-338-3p, miR-223, miR-148a and miR-107 in early stage breast cancer remain unclear. Studies examining the function of these miRNAs in breast cancer have primarily been performed on different breast cancer cell lines with varying results [32]–[35]. Elevated levels of miR-223 in breast cancer cells has been shown to be associated with decreased migration, increased cell death in anoikis conditions and augmented sensitivity to chemotherapy [32]. Further, miR-223 has been reported to be down-regulated in whole blood from patients with Luminal A breast cancer compared to healthy controls [36], and in a recent study by our group, miR-107 was up-regulated in the serum of patients with early stage breast cancer compared to healthy controls [37]. However, the impact of these specific miRNAs on breast cancer progression or recurrence remains to be determined. Evaluation of whether these specific miRNAs are useful potential biomarkers in follow-up of post-surgical early stage breast cancer patients requires studies using blood samples consecutively collected during long term follow-up.

A major concern when analyzing circulating miRNAs is the normalization procedure. In our study, we normalized to the mean Cp-value of all samples, which is recommended when dealing with multiple assays [28]. However, this method is not applicable when analyzing only a few miRNAs. Alternatively, the most stably and consistently expressed miRNAs may be used for normalization. Most studies have used miR-16 for endogenous normalization, but recent studies have found miR-16 to be unsuitable as reference [25], [38].

In conclusion, we identified a panel of 4 circulating miRNAs that may be used to monitor the presence of microscopic residual disease in breast cancer, findings that should be validated in a larger prospective study, prefe+rably with several paired samples at different time points, to determine their potential as clinical biomarkers. Moreover, further work to develop standards for circulating miRNA studies, including sample preparation, control for sample hemolysis and especially normalization of measured Cp-values, should also be performed.
